# Chronic low back pain and postural instability: interaction effects of pain severity, age, BMI, and disability

**DOI:** 10.3389/fpubh.2025.1497079

**Published:** 2025-01-17

**Authors:** Adel Alshahrani, Ravi Shankar Reddy, Sunil Kumar Ravi

**Affiliations:** ^1^Department of Medical Rehabilitation Sciences, College of Applied Medical Sciences, Najran University, Najran, Saudi Arabia; ^2^Department of Medical Rehabilitation Sciences, College of Applied Medical Sciences, King Khalid University, Abha, Saudi Arabia

**Keywords:** postural balance, body mass index, pain measurement, disability, low back pain

## Abstract

**Objectives:**

This study aimed to (1) compare postural sway patterns between individuals with chronic low back pain (CLBP) and asymptomatic controls, (2) evaluate correlations between pain severity and postural stability variables, and (3) assess the interaction effects of age, BMI, pain severity, and disability on postural stability under eyes-open and eyes-closed conditions.

**Methods:**

Postural stability (sway area, sway velocity, CoP displacement) was assessed in 88 CLBP patients and 88 controls using a stabilometric platform. Pain severity (VAS) and disability (ODI) were recorded alongside demographic data. Statistical analyses included *t*-tests, Pearson’s correlations, and ANOVA to explore group differences, correlations, and interaction effects.

**Results:**

Chronic low back pain patients exhibited significantly greater postural sway across all conditions, with larger sway area (16.80 ± 6.10 cm^2^ vs. 11.50 ± 4.10 cm^2^, *p* = 0.004) and higher sway velocity (4.10 ± 1.40 cm/s vs. 2.90 ± 1.00 cm/s, *p* = 0.009) under eyes-closed conditions. Pain severity correlated with sway velocity (*r* = 0.52, *p* = 0.003) and CoP displacement (*r* = 0.57, *p* = 0.002). Interaction effects indicated greater instability in older, obese individuals with severe pain and high disability.

**Conclusion:**

Chronic low back pain is associated with impaired postural stability, influenced by pain severity, BMI, age, and disability. Targeted interventions addressing these factors are essential for improving balance and reducing fall risk.

## Introduction

Chronic low back pain (CLBP) is a widespread musculoskeletal disorder that impacts millions of people globally (1). It is a major contributor to disability, diminished quality of life, and escalated healthcare expenses. CLBP is defined by ongoing or recurrent pain and discomfort in the lumbar area that persists for 12 weeks or more (1, 2). CLBP can result from various causes, including degenerative changes, muscle strain, or nerve compression, and often lacks a clear underlying pathology, making it difficult to manage effectively (3). Due to its chronic nature, CLBP not only impairs physical function but also impacts emotional well-being, contributing to anxiety, depression, and social isolation (3). As a result, CLBP patients experience limitations in their ability to perform daily activities, often requiring long-term medical interventions, rehabilitation, or even surgical procedures (4). The widespread prevalence and multifactorial nature of CLBP underscore the need for a deeper understanding of its effects on physical function, particularly in relation to postural stability, which plays a crucial role in maintaining balance and preventing falls (5).

Postural stability is the capacity to keep the body’s center of mass within its base of support, ensuring balance during both static and dynamic movements (6, 7). In individuals with CLBP, postural control is often compromised due to pain-related alterations in motor control and proprioceptive function (8). Several key postural stability variables, including sway area, sway velocity, and center of pressure (CoP) displacement, have been used to quantify postural instability in this population (9). Sway area measures the extent of movement within the base of support, while sway velocity refers to the speed of postural adjustments, and CoP displacement indicates the overall movement of the body’s center of pressure relative to the support surface (10). These variables provide valuable insights into the stability and balance of individuals with larger sway areas, higher sway velocities, and greater CoP displacement, indicating poorer postural control (10). Previous studies have demonstrated that CLBP patients exhibit greater sway in comparison to asymptomatic individuals, particularly under challenging sensory conditions such as eyes-closed or single-leg stance tasks (11). These postural instability variables are often exacerbated by factors such as increased pain severity and functional disability, highlighting the potential link between pain, sensory processing, and motor control deficits in individuals with CLBP (12).

The interaction effects of age, body mass index (BMI), pain severity, and disability on postural stability are of particular relevance to CLBP patients (13). Aging is associated with a natural decline in muscle strength, joint flexibility, and sensory processing, which can impair postural control and increase the risk of falls (14, 15). When combined with the pain and dysfunction associated with CLBP, these age-related changes can significantly worsen postural stability, leading to greater sway area, velocity, and CoP displacement (16). BMI, as an indicator of obesity or overweight, is another critical factor influencing postural control (17). Increased body mass can alter biomechanics, affecting the distribution of forces across the joints and muscles and leading to compensatory movements that further destabilize posture (17). Pain severity, particularly in CLBP, can disrupt normal neuromuscular coordination and proprioceptive feedback, resulting in impaired postural adjustments (18). Additionally, disability, as measured by the Oswestry Disability Index (ODI), reflects the extent to which pain and functional limitations impact an individual’s daily life (19). Higher ODI scores are associated with greater functional impairment, which can further compromise the ability to maintain postural control (20). Therefore, understanding the combined impact of these factors on postural stability is critical for developing targeted interventions to reduce fall risk and improve balance in CLBP patients.

Despite growing recognition of the importance of postural stability in CLBP, there remains a need for more comprehensive research that evaluates the combined effects of age, BMI, pain severity, and disability on postural control. While several studies have investigated individual factors affecting postural stability, few have explored how these variables interact with one another in a population of CLBP patients (21). Most studies tend to focus on either age or pain severity in isolation, overlooking the potential synergies between multiple contributing factors (22, 23). Furthermore, much of the research has been conducted in controlled laboratory settings using single-task balance assessments, which may not fully capture the complexities of postural instability experienced by CLBP patients in real-world environments (24). Additionally, existing studies often fail to address how sensory conditions, such as eyes open versus eyes closed, further challenge postural control in this population (24). Given the multifactorial nature of postural control, it is essential to investigate these interaction effects in a more holistic manner to better understand the underlying mechanisms contributing to postural instability in CLBP patients. Identifying these interaction effects is particularly important for developing tailored rehabilitation programs that address the specific needs of patients based on their age, BMI, pain levels, and disability status. While previous studies have investigated individual factors such as age or pain severity in isolation, there is a significant gap in understanding how these factors interact to influence postural stability in individuals with CLBP. This study aims to address this gap by comprehensively evaluating the combined effects of age, BMI, pain severity, and disability, providing a novel perspective that is critical for developing targeted, multifactorial rehabilitation strategies.

The current study seeks to fill this research gap by comprehensively evaluating postural stability variables in individuals with CLBP and asymptomatic controls under varying sensory conditions. Specifically, the study aims to (1) compare postural sway patterns between CLBP patients and asymptomatic controls using real-time stabilometric force platform data during static postural tasks, (2) assess the correlation between pain severity and postural stability variables in the CLBP group, and (3) investigate the interaction effects of age, BMI, pain severity, and disability on postural stability under eyes-open versus eyes-closed conditions. It is hypothesized that individuals with CLBP will exhibit significantly greater postural sway compared to asymptomatic controls and that higher pain severity, greater BMI, and increased disability will be associated with more pronounced postural instability. Moreover, it is expected that these effects will be more pronounced in older adults and under eyes-closed conditions, where postural control relies more heavily on proprioceptive feedback.

## Methods

### Study design and ethics

This cross-sectional study was conducted between April 2023 and February 2024 at the Department of Orthopedics and Rehabilitation, King Khalid University Hospital, a tertiary care center specializing in musculoskeletal disorders. Ethical approval was obtained from the hospital’s Institutional Review Board (REC#643–2023) on 24/03/2023. Written informed consent was obtained from all participants prior to their inclusion. To ensure participant safety during stability assessments, all tasks were conducted under the supervision of trained professionals in a controlled environment. Participants were instructed to perform the tasks at their comfort level, with the option to stop at any time if discomfort or instability occurred. Additionally, a safety harness system was available to prevent falls during assessments, and participants were closely monitored throughout each trial.

### Participants

Participants in this study were recruited using a convenience sampling method from the outpatient clinics of the Department of Orthopedics and Rehabilitation. The study included individuals diagnosed with CLBP and asymptomatic controls. CLBP was defined as pain localized in the lumbar region, persisting for at least 12 weeks, in accordance with clinical guidelines for the diagnosis and management of low back pain (25). Participants with CLBP were included if they were between 18 and 65 years of age, had experienced non-specific low back pain without radiculopathy or significant neurological deficits, and were medically stable enough to undergo postural stability testing. The asymptomatic control group consisted of individuals with no history of low back pain, musculoskeletal disorders, or neurological conditions affecting posture or balance.

The inclusion criteria for the CLBP group required participants to have a clinical diagnosis of CLBP confirmed by a physician based on history, physical examination, and imaging studies. All participants were required to have a Visual Analogue Scale (VAS) pain score of 4 or higher at least once in the preceding 3 months to ensure that the pain was sufficiently symptomatic. The asymptomatic control group was included based on the absence of any history of low back pain or postural instability, with participants matched to the CLBP group by age and body mass index (BMI) where possible. Exclusion criteria for both groups included the presence of any significant neurological, vestibular, or orthopedic conditions that could independently affect balance, such as stroke, multiple sclerosis, severe lower limb injuries, or vestibular dysfunction. Additionally, individuals with previous spinal surgery, acute back injuries, or systemic conditions like uncontrolled diabetes or cardiovascular diseases were excluded. Pregnant individuals, those unable to provide informed consent, and those undergoing active treatment for severe psychiatric disorders were also excluded from participation.

### Postural stability assessment

**Figure 1:**
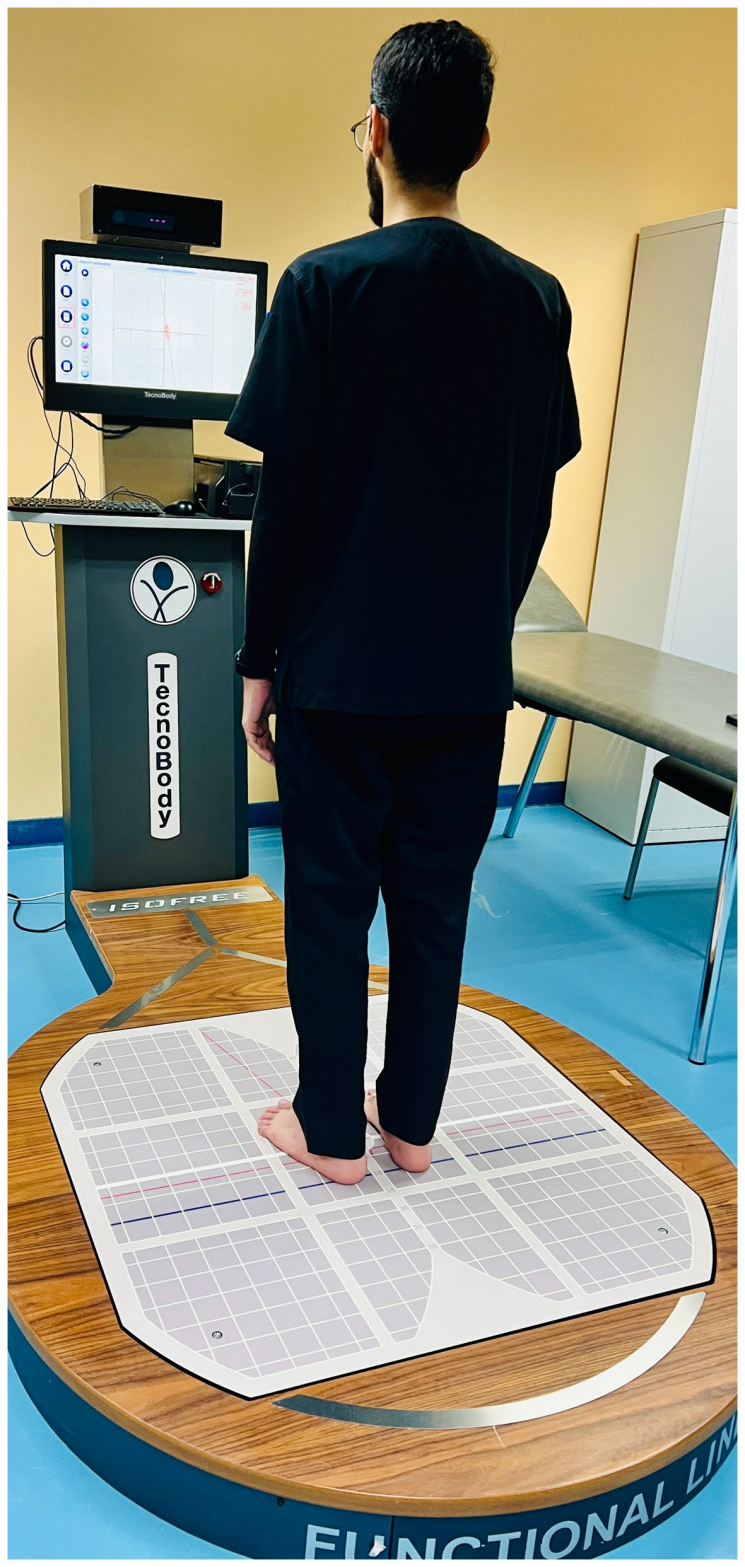
Assessment of postural stability using computerized posturography stabilometric force platform system. “Illustration of the postural stability assessment setup showing the stabilometric platform and sensory conditions (eyes open and eyes closed) used to measure sway area, sway velocity, and CoP displacement.”

The primary outcomes of this study were the postural stability variables, including sway area (cm^2^), sway velocity (cm/s), and center of pressure (CoP) displacement (mm), measured using a computerized posturographic stabilometric platform system (26). This system is widely regarded as reliable and highly accurate for assessing overall postural stability (26). The equipment was carefully calibrated before each testing session to ensure precision, and all participants were instructed to wear loose-fitting garments to minimize any restrictions to movement during the assessment (27). Each participant stood barefoot on the stabilometric platform system, which recorded real-time data on postural sway during static tasks. Postural stability was assessed under two different sensory conditions: eyes open and eyes closed (Figure 1) (27). During the eyes-open condition, participants were instructed to focus on a fixed point in the computer monitor in front of them, while during the eyes-closed condition, they were asked to maintain balance without any visual feedback. Each postural stability task was conducted for 30 s per trial, with participants performing three trials under each sensory condition (eyes open and eyes closed). For each task, participants were guided by auditory instructions from the posturographic system. To ensure accuracy and reliability, each postural task was performed three times, and the best performance (i.e., the trial with the most stable variables) was selected for analysis.

### Sample size calculation

The sample size for this study was calculated using G*Power statistical software (28), aiming for a power of 0.80, an alpha level of 0.05, and a moderate effect size (Cohen’s *d* = 0.5). Based on these parameters, a minimum of 88 participants per group (chronic low back pain and asymptomatic controls) was determined to be sufficient for detecting significant differences in postural stability variables and for performing the required statistical analyses, including independent t-tests and repeated measures ANOVA.

### Data analysis

The data conformed to a normal distribution, as verified by the Shapiro–Wilk test for normality, and the homogeneity of variances was evaluated using Levene’s test. Statistical analysis was conducted via SPSS software, with a *p*-value below 0.05 deemed statistically significant. Two-sample independent t-tests were used to compare postural sway variables (sway area, sway velocity, and CoP displacement) between individuals with CLBP and asymptomatic controls. Pearson’s correlation was employed to analyze the relationship between pain severity (measured by VAS and ODI) and postural stability variables in the CLBP group. Additionally, a three-way repeated measures ANOVA was utilized to explore the interaction effects of age, BMI, pain severity, and disability on postural stability under varying sensory conditions (eyes open vs. eyes closed). Effect sizes for t-tests were determined using Cohen’s *d*, while partial eta-squared (*η*^2^) was used for ANOVA.

## Results

The demographic and clinical characteristics of participants in the CLBP group and control group showed no significant differences in age or sex distribution (Table 1). However, the CLBP group had a significantly higher body mass index (BMI) compared to the asymptomatic group. Participants in the CLBP group reported an average pain severity of 6.30 (VAS) and an average Oswestry Disability Index (ODI) score of 34.50%. Both groups were similar in terms of physical activity levels, smoking status, and employment status, with no statistically significant differences in these variables.

**Table 1:** Demographic and clinical characteristics of study participants (*n* = 176).

Characteristic	CLBP	Asymptomatic	*p*-value
Age (years), mean ± SD	45.60 ± 10.300	43.90 ± 9.800	0.350
Sex, *n* (%)			
Male	46 (52.273%)	48 (54.545%)	0.750
Female	42 (47.727%)	40 (45.455%)	
BMI (kg/m^2^), mean ± SD	27.20 ± 3.800	25.80 ± 3.100	0.023
Duration of CLBP (years), mean ± SD	7.50 ± 4.300	–	–
Pain Severity (VAS), mean ± SD	6.30 ± 1.400	–	–
ODI Score (%, mean ± SD)	34.50 ± 15.200	–	–
Physical activity level			
Low, *n* (%)	35 (39.773%)	28 (31.818%)	0.220
Moderate, *n* (%)	45 (51.136%)	50 (56.818%)	0.490
High, *n* (%)	8 (9.091%)	10 (11.364%)	0.610
Smoking status, *n* (%)			
Smoker	20 (22.727%)	15 (17.045%)	0.370
Non-smoker	68 (77.273%)	73 (82.955%)	
Work status (employed), *n* (%)	50 (56.818%)	55 (62.500%)	0.470
Work status (Unemployed), *n* (%)	38 (43.182%)	33 (37.500%)	0.470

CLBP, chronic low back pain; BM, body mass index; VAS, Visual Analogue Scale; ODI, Oswestry disability index; SD, standard deviation.

**Figure 2:**
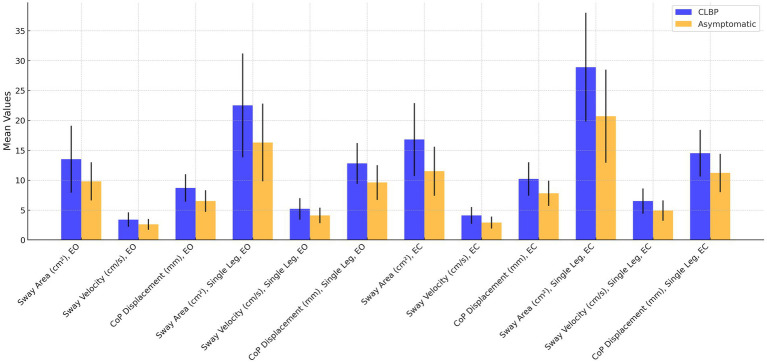
Comparison of postural stability metrics between individuals with chronic low back pain (CLBP) and asymptomatic controls under various conditions. Metrics include Sway Area (cm^2^), Sway Velocity (cm/s), and Center of Pressure (CoP) Displacement (mm) measured with eyes open (EO), eyes closed (EC), and single-leg stance. Error bars represent standard deviations.

The comparison of postural sway variables between individuals with CLBP and asymptomatic controls demonstrated significant differences across several measures (Table 2 and Figure 2). In the eyes-open condition, the CLBP group had a larger sway area (13.50 ± 5.60 cm^2^) compared to the controls (9.80 ± 3.20 cm^2^), with a mean difference of 3.70 cm^2^ (*p* = 0.012, Cohen’s *d* = 0.66). This difference increased in the eyes-closed condition, where the CLBP group showed a sway area of 16.80 ± 6.10 cm^2^ compared to 11.50 ± 4.10 cm^2^ in controls, with a mean difference of 5.30 cm^2^ (*p* = 0.004, Cohen’s *d* = 0.80). Sway velocity was also significantly higher in the CLBP group, with values of 3.40 ± 1.20 cm/s (eyes open) and 4.10 ± 1.40 cm/s (eyes closed), compared to 2.60 ± 0.90 cm/s and 2.90 ± 1.00 cm/s in the control group, respectively (*p* = 0.019 and *p* = 0.009). Similarly, CoP displacement was greater in the CLBP group under both eyes-open (8.70 ± 2.30 mm vs. 6.50 ± 1.80 mm, *p* = 0.008, Cohen’s *d* = 0.78) and eyes-closed conditions (10.20 ± 2.80 mm vs. 7.80 ± 2.10 mm, *p* = 0.005, Cohen’s *d* = 0.85). These differences were further amplified during the single-leg stance, with the CLBP group showing significantly larger sway area, sway velocity, and CoP displacement compared to the control group, indicating substantial postural instability.

**Table 2:** Comparison of postural sway variables between CLBP and asymptomatic groups (*n* = 166).

Postural sway variables	CLBP	Asymptomatic	Mean difference, (95% CI)	Effect size (Cohen’s *d*)	*p*-value
Sway area (cm^2^), EO	13.50 ± 5.60	9.80 ± 3.20	3.70 (1.20–6.20)	0.66	0.012
Sway velocity (cm/s), EO	3.40 ± 1.20	2.60 ± 0.90	0.80 (0.20–1.40)	0.58	0.019
CoP displacement (mm), EO	8.70 ± 2.30	6.50 ± 1.80	2.20 (1.00–3.40)	0.78	0.008
Sway area (cm^2^), single leg, EO	22.50 ± 8.70	16.30 ± 6.50	6.20 (3.10–9.30)	0.82	0.002
Sway velocity (cm/s), single leg, EO	5.20 ± 1.80	4.10 ± 1.30	1.10 (0.50–1.70)	0.64	0.015
CoP displacement (mm), single leg, EO	12.80 ± 3.40	9.60 ± 2.90	3.20 (1.50–4.90)	0.70	0.010
Sway area (cm^2^), EC	16.80 ± 6.10	11.50 ± 4.10	5.30 (2.50–8.10)	0.80	0.004
Sway velocity (cm/s), EC	4.10 ± 1.40	2.90 ± 1.00	1.20 (0.50–1.90)	0.72	0.009
CoP displacement (mm), EC	10.20 ± 2.80	7.80 ± 2.10	2.40 (1.20–3.60)	0.85	0.005
Sway area (cm^2^), single leg, EC	28.90 ± 9.10	20.70 ± 7.80	8.20 (4.90–11.50)	0.89	0.001
Sway velocity (cm/s), single leg, EC	6.50 ± 2.10	4.90 ± 1.70	1.60 (0.80–2.40)	0.76	0.007
CoP displacement (mm), single leg, EC	14.50 ± 3.90	11.20 ± 3.20	3.30 (1.80–4.80)	0.73	0.009

CLBP, chronic low back pain; EO, eyes open; EC, eyes closed; SD, standard deviation; CoP, center of pressure; CI, confidence interval. Effect size conventions: for Cohen’s *d*, small = 0.2, moderate = 0.5, large = 0.8. For partial eta-squared (𝜂^2^), small = 0.01, moderate = 0.06, large = 0.14.

**Figure 3:**
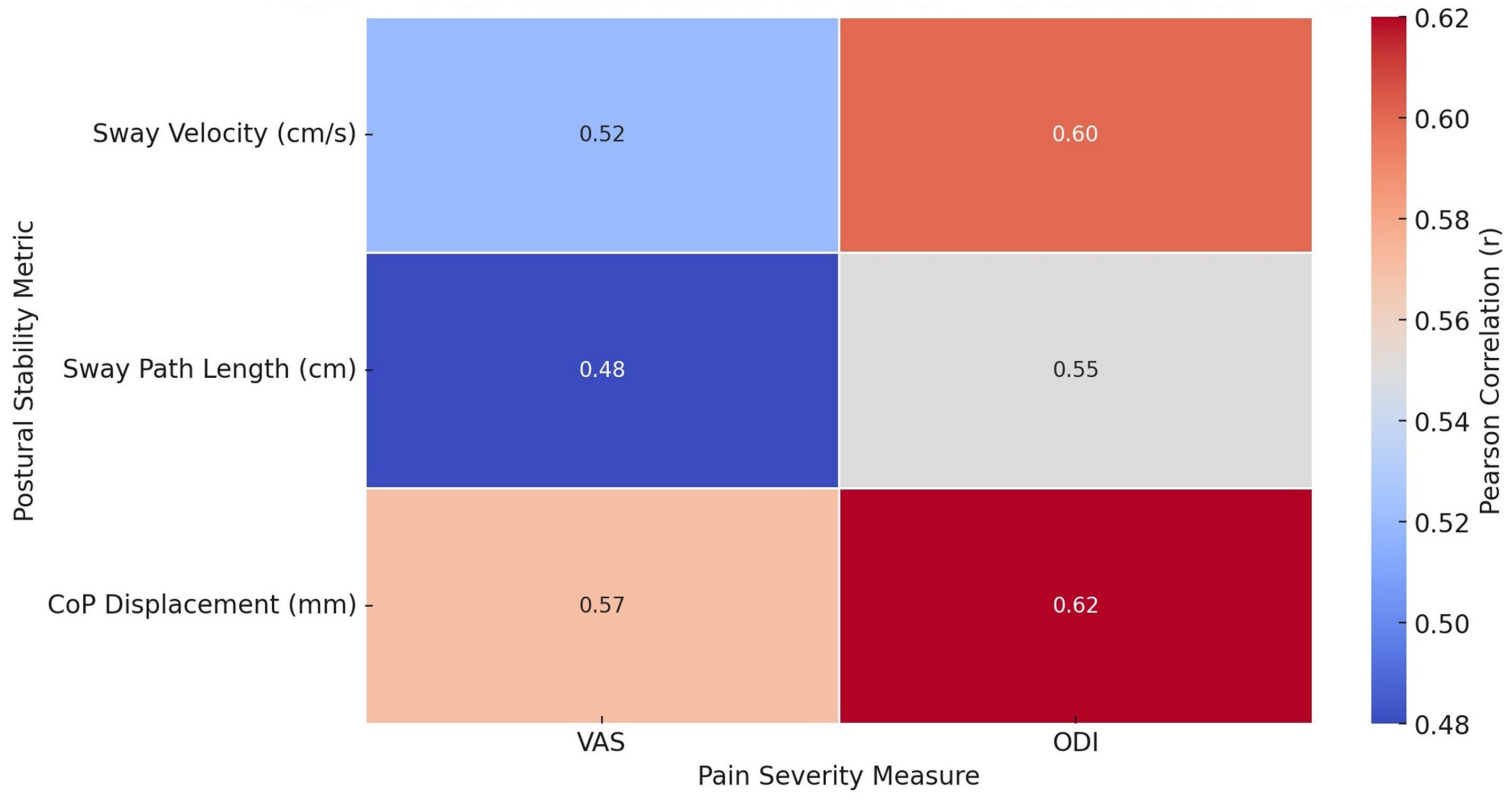
Heatmap illustrating Pearson correlation coefficients (*r*) between pain severity measures (VAS and ODI) and postural stability metrics (Sway Velocity, Sway Path Length, and CoP Displacement). Stronger correlations are highlighted in warmer colors.

Significant positive correlations were observed between pain severity and postural stability variables in individuals with CLBP (Figure 3). Higher pain severity, as measured by the Visual Analogue Scale (VAS), was moderately correlated with increased sway velocity (*r =* 0.52, *p =* 0.003), sway path length (*r =* 0.48, *p =* 0.007), and center of pressure (CoP) displacement (*r =* 0.57, *p =* 0.002). Similarly, disability severity, measured by the Oswestry Disability Index (ODI), was strongly correlated with postural instability variables, showing higher correlations with sway velocity (*r* = 0.60, *p* = 0.001), sway path length (*r =* 0.55, *p =* 0.002), and CoP displacement (*r =* 0.62, *p =* 0.001).

**Figure 4:**
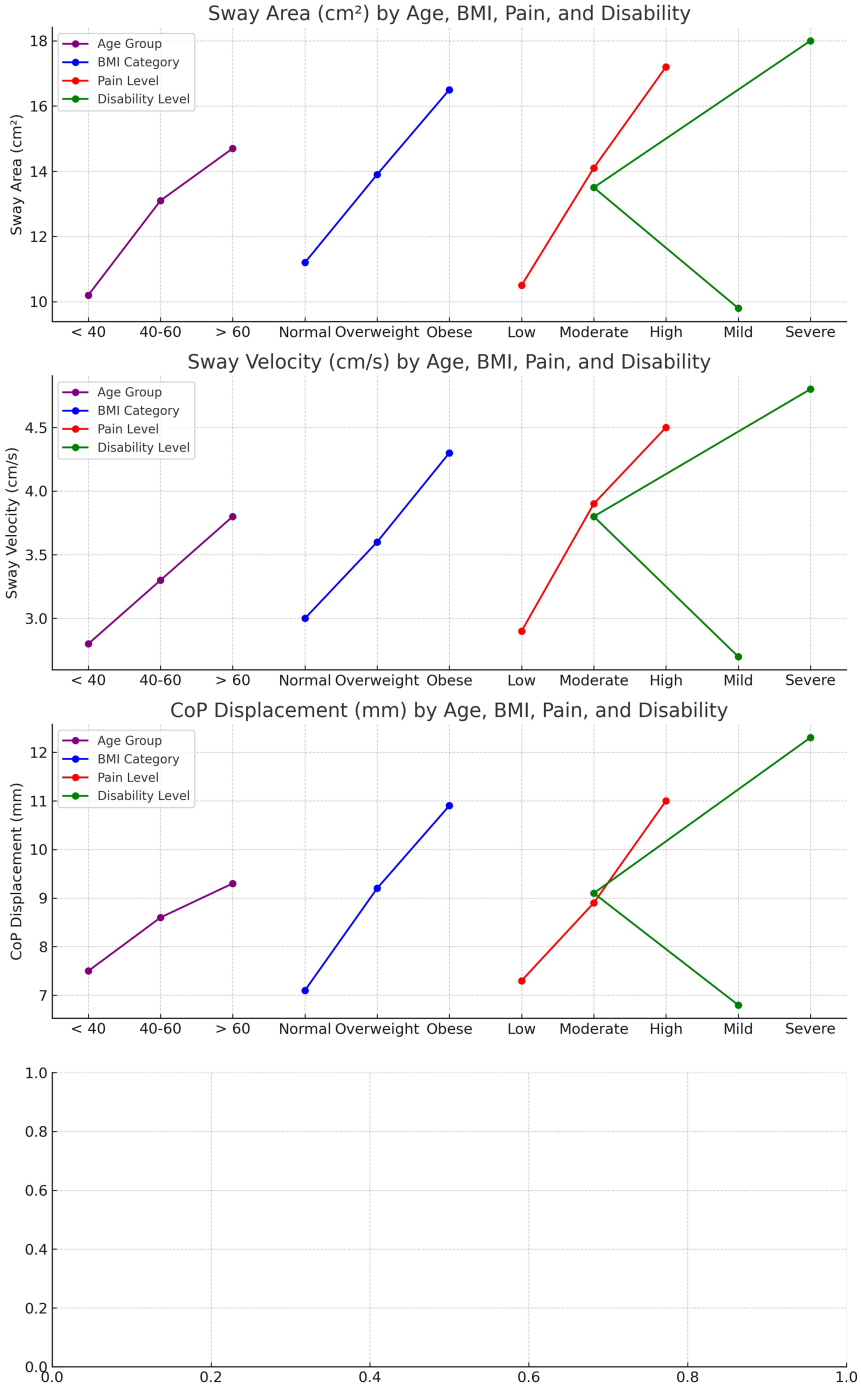
Interaction effects of age, BMI, pain severity, and disability on postural stability metrics in CLBP patients. Sway Area, Sway Velocity, and CoP Displacement all increase with higher BMI, greater pain severity, and higher disability levels, with older individuals showing the greatest instability across these metrics. The trends highlight the compounded impact of these factors on postural control.

Significant interaction effects were observed between age, body mass index (BMI), pain severity, and disability on postural stability variables in individuals with CLBP (Table 3 and Figure 4). Across all age groups, higher BMI, elevated pain levels, and greater disability were associated with increased postural instability. Younger participants (<40 years) with obesity and severe pain showed the largest sway area (16.50 cm^2^ eyes open, 20.20 cm^2^ eyes closed) and sway velocity (4.10 cm/s eyes open, 4.90 cm/s eyes closed), as well as increased center of pressure (CoP) displacement (10.30 mm eyes open, 12.10 mm eyes closed). Similarly, older participants (>60 years) with obesity and high pain severity exhibited the greatest instability, with sway area reaching 19.80 cm^2^ (eyes open) and 23.50 cm^2^ (eyes closed), and CoP displacement of 12.00 mm and 13.70 mm under eyes open and closed conditions, respectively. These findings highlight the compounded effect of age, BMI, pain, and disability on postural control in CLBP patients.

**Table 3:** Interaction effects of age, BMI, pain severity, and disability on postural stability variables.

Age	BMI	Pain level	Disability level	Sway area (cm^2^), EO	Sway area (cm^2^), EC	Sway velocity (cm/s), EO	Sway velocity (cm/s), EC	CoP displacement (mm), EO	CoP displacement (mm), EC	*p*-value
<40	Normal	Low	Mild	10.20	12.80	2.80	3.30	7.50	8.90	0.023
<40	Obese	High	Severe	16.50	20.20	4.10	4.90	10.30	12.10	0.005
40–60	Overweight	Moderate	Moderate	13.10	16.40	3.30	4.00	8.60	10.10	0.032
40–60	Obese	High	Severe	18.40	21.30	4.40	5.10	11.20	13.00	0.008
>60	Overweight	Moderate	Moderate	14.70	18.20	3.80	4.40	9.30	11.00	0.014
>60	Obese	High	Severe	19.80	23.50	4.70	5.50	12.00	13.70	0.001

BMI, body mass index; EO, eyes open; EC, eyes closed; CoP, center of pressure.

## Discussion

The present study aimed to evaluate postural stability differences between individuals with CLBP and asymptomatic controls, explore the relationship between pain severity and postural stability in CLBP patients, and assess the interaction effects of age, BMI, pain severity, and disability on postural control. The results demonstrated that CLBP patients exhibited significantly greater postural instability across multiple variables, including sway area, sway velocity, and center of pressure (CoP) displacement, compared to asymptomatic controls, particularly under eyes-closed and single-leg stance conditions. Additionally, a moderate to strong positive correlation was found between pain and disability severity and increased postural instability, suggesting that greater pain and functional impairment are associated with worsened balance. The interaction analysis further revealed that older age, higher BMI, and greater pain and disability levels collectively exacerbated postural instability, particularly in challenging sensory conditions, emphasizing the multifactorial nature of balance impairments in CLBP patients.

The results indicate that individuals with CLBP exhibit significantly greater postural instability compared to asymptomatic controls across various postural sway variables. These differences could be attributed to the altered motor control strategies and proprioceptive deficits often observed in CLBP patients (8). Pain and discomfort in the lower back may lead to compensatory movements and reduced muscle activation in the core and lower extremities, contributing to impaired balance (21). Additionally, postural control is more challenged in the eyes-closed and single-leg stance conditions, as the absence of visual input and decreased base of support place greater reliance on proprioceptive feedback, which may be diminished in CLBP individuals (29). These results suggest that CLBP patients may have difficulty maintaining postural stability, especially when subjected to challenging sensory conditions (29). Previous studies support these findings, demonstrating a consistent association between chronic pain and impaired balance (11, 30). Nogueira et al. (11) found that individuals with CLBP showed significantly larger sway areas and higher sway velocities than controls, particularly under eyes-closed conditions (11). Similarly, Mohammadi et al. (31) reported that CLBP patients exhibited decreased proprioceptive accuracy, leading to impaired postural control (31). These studies reinforce the notion that sensory deficits and altered motor control mechanisms in CLBP patients contribute to their increased postural instability, particularly in tasks that require greater sensorimotor integration, such as single-leg stance and eyes-closed conditions (31).

The results demonstrate that higher levels of pain and disability in individuals with CLBP are significantly associated with increased postural instability. The positive correlations between Visual Analogue Scale (VAS) scores and postural sway variables, including sway velocity, sway path length, and center of pressure (CoP) displacement, suggest that as pain severity increases, patients experience more difficulty maintaining stable posture (32). This instability is further exacerbated by higher disability levels, as measured by the Oswestry Disability Index (ODI), which showed even stronger correlations with postural sway variables (33). This could be due to the compounding effects of pain and reduced functional ability, leading to altered movement strategies, impaired proprioception, and muscle weakness in CLBP patients (34). These factors likely impair the body’s ability to regulate balance, particularly under conditions that require increased sensorimotor coordination (35). Previous studies have consistently demonstrated similar findings, reinforcing the link between pain, disability, and postural instability in CLBP patients (13). Shanbehzadeh et al. (36) and Sung and Lee (37) found that increased pain and disability in CLBP patients contributed to larger sway areas and higher sway velocities, indicating reduced postural control(36, 37). In particular, their research highlighted that pain interferes with proprioceptive feedback mechanisms, which are essential for maintaining balance (37). Moreover, Yap et al. (38) emphasized that disability severity correlates with greater postural sway, further supporting the present findings (36, 38). These studies align with the observed correlations between higher VAS and ODI scores and impaired postural stability, suggesting that pain and disability act as significant contributors to balance dysfunction in CLBP populations (38).

The interaction effects between age, body mass index (BMI), pain severity, and disability on postural stability in individuals with CLBP highlight that these factors collectively exacerbate postural instability. As observed, younger participants (<40 years) with obesity and high pain levels exhibited the greatest postural sway, particularly in more challenging conditions like eyes-closed or single-leg stance (39). This may be due to the reduced ability of the musculoskeletal and sensory systems to compensate for impaired proprioception and pain in CLBP patients (40), leading to altered motor strategies and increased reliance on visual cues to maintain balance (31, 40). Similarly, older adults (>60 years) with elevated BMI, severe pain, and higher disability scores displayed even greater postural instability, suggesting that aging-related declines in muscle strength, joint stability, and proprioceptive feedback further aggravate the effects of CLBP on balance control (41). Previous research supports these findings, demonstrating the multifactorial impact of age, BMI, pain, and disability on postural stability in CLBP patients (41). Kummer et al. (42) have emphasized that chronic pain alters motor control, resulting in compensatory movement patterns that disrupt postural regulation. Moreover, studies by Tallis et al. (43) and Viseux et al. (44) have highlighted the role of obesity and aging in worsening postural control, particularly when coupled with pain and disability (44). These studies align with the current results, showing that higher BMI and pain severity in CLBP patients leads to greater postural instability, especially under conditions that require heightened sensorimotor integration, such as eyes-closed tasks (43, 44). Overall, the interaction between these factors reinforces the complex nature of balance impairments in CLBP and underscores the importance of targeting these variables in rehabilitation strategies (43, 44). Obesity appears to exacerbate postural instability in CLBP patients due to its biomechanical and physiological effects (45). Increased body mass shifts the center of gravity forward, placing additional strain on the lumbar spine and lower extremity joints, which are already compromised in individuals with CLBP (45). These altered biomechanics may necessitate compensatory motor strategies that further destabilize posture. Additionally, obesity is associated with systemic inflammation and impaired neuromuscular function, which can negatively affect proprioceptive feedback and motor control, key components of postural stability (46). These findings highlight the critical need for integrated rehabilitation strategies that combine weight management with targeted sensorimotor training to mitigate these effects. By addressing obesity-related factors, clinicians can potentially reduce fall risk and improve functional outcomes in this population.

### Clinical significance

The clinical significance of this study lies in its identification of key factors contributing to postural instability in individuals with CLBP, particularly under challenging sensory conditions. The findings emphasize that elevated pain severity, higher body mass index (BMI), and greater disability levels exacerbate balance impairments, with these effects being more pronounced in older adults (47). This highlights the need for targeted rehabilitation strategies that address not only pain management but also weight reduction and functional mobility improvements to mitigate postural instability. Understanding the compounded impact of age, BMI, pain, and disability on postural control provides valuable insight for clinicians in developing comprehensive, individualized treatment plans aimed at reducing fall risk and enhancing the overall quality of life in CLBP patients. Furthermore, the study underscores the importance of incorporating balance training and sensorimotor exercises in therapeutic interventions to improve postural control in this population.

### Limitations and areas of future research

Although the study provides valuable insights into the interaction effects of age, BMI, pain severity, and disability on postural instability in CLBP patients, certain limitations should be noted. The use of convenience sampling, while practical for recruiting participants from a clinical population, may introduce selection bias and limit the representativeness of the sample. Consequently, the findings may not fully generalize to broader populations, particularly those with different socioeconomic, cultural, or activity-level characteristics. Furthermore, the controlled environment of postural assessments may not entirely reflect real-world conditions, potentially reducing the external validity of the results. Future studies should aim to use random sampling methods and include diverse populations to enhance the generalizability of findings. Additionally, incorporating ecologically valid assessment protocols that mimic everyday activities would provide deeper insights into the functional implications of postural instability in CLBP patients.

## Conclusion

This study demonstrates that individuals with CLBP experience significantly greater postural instability compared to asymptomatic controls, particularly under challenging sensory conditions such as eyes-closed and single-leg stances. The findings highlight that higher pain severity, increased body mass index (BMI), and greater disability levels are strongly associated with impaired postural control, with the effects being more pronounced in older adults. These results underscore the importance of addressing not only pain management but also factors such as weight and functional capacity in the clinical management of CLBP patients. The study emphasizes the need for comprehensive rehabilitation strategies that incorporate balance training and sensorimotor exercises to reduce fall risk and improve overall stability in this population.

## Data Availability

The datasets presented in this study can be found in online repositories. The names of the repository/repositories and accession number(s) can be found in the article/Supplementary material.
